# Sea snake cathelicidin (Hc-cath) exerts a protective effect in mouse models of lung inflammation and infection

**DOI:** 10.1038/s41598-019-42537-8

**Published:** 2019-04-15

**Authors:** Simon R. Carlile, Jenna Shiels, Lauren Kerrigan, Rebecca Delaney, Julianne Megaw, Brendan F. Gilmore, Sinéad Weldon, John P. Dalton, Clifford C. Taggart

**Affiliations:** 1 Airway Innate Immunity Research (AiiR) Group, Centre for Experimental Medicine, The Wellcome-Wolfson Institute for Experimental Medicine, School of Medicine, Dentistry and Biomedical Sciences, Queen’s University Belfast, 97 Lisburn Road, Belfast, BT9 7BL Northern Ireland UK; 2 School of Biological Sciences, Queen’s University Belfast, 97 Lisburn Road, Belfast, BT9 7BL Northern Ireland UK; 3 School of Pharmacy, Queen’s University Belfast, 97 Lisburn Road, Belfast, BT9 7BL Northern Ireland UK

## Abstract

We investigated the anti-inflammatory and antibacterial activities of Hc-cath, a cathelicidin peptide derived from the venom of the sea snake, *Hydrophis cyanocyntus*, using *in vivo* models of inflammation and infection. Hc-cath function was evaluated in *in vitro*, *in vivo* in the wax moth, *Galleria mellonella*, and in mouse models of intraperitoneal and respiratory *Pseudomonas aeruginosa* infection. Hc-Cath downregulated LPS-induced pro-inflammatory responses in macrophages and significantly improved the survival of *P. aeruginosa* infected *G. mellonella* over a 5-day period. We also demonstrated, for the first time, that Hc-cath can modulate inflammation in a mouse model of LPS-induced lung inflammation by significantly reducing the release of the pro-inflammatory cytokine and neutrophil chemoattractant, KC, resulting in reduced cellular infiltration into the lungs. Moreover, Hc-cath treatment significantly reduced the bacterial load and inflammation in mouse models of *P. aeruginosa* intraperitoneal and respiratory infection. The effect of Hc-cath in our studies highlights the potential to develop this peptide as a candidate for therapeutic development.

## Introduction

Respiratory infections represent a major clinical burden globally and have major implications for patients with underlying health conditions such as cystic fibrosis (CF), where bacterial infection can exacerbate pre-existing inflammation. *Pseudomonas aeruginosa* infection is particularly difficult to treat in CF patients, due to its ability to form biofilms with antibiotic tolerant persister cells, in addition to the bacteria’s ability to acquire antibiotic resistance^1^. The World health organisation (WHO) have adopted a five-point plan to combat the rise of antibiotic resistance including the development of new classes of antibiotics^2^.

Antimicrobial peptides represent a largely untapped resource of new antibiotic agents that could supplement current antibiotics. This study focuses on a group of antimicrobial proteins known as cathelicidins. The proteins in this family are grouped on the basis of their shared structure consisting of a pro-form conjugated to a conserved cathelin domain, which is cleaved to release an active C-terminal peptide^3^. The only known human cathelicidin, CAP18, is a host defense protein secreted predominantly by neutrophils. C-terminal cleavage releases a 37-amino acid peptide, LL-37, which plays an important role in the innate immune system. LL-37 displays a broad range of antimicrobial activities including antifungal, antiviral and antibacterial activity^4^. Bacteria such as *Neisseria gonorrhoea*^5^ and *Shigella*^6^ can downregulate the expression of LL-37, increasing their infectivity. The absence of LL-37 in the neutrophils of patients with Morbus Kostmann syndrome leads to increased rates of periodontitis^7^. The antimicrobial activity of LL-37 is impaired in CF patients due to the reduced airway pH^8^. In addition, LL-37 activity can be affected by binding to DNA and glycosaminoglycans^9^ or from degradation by proteases in CF sputum^10^.

Cathelicidins are also found in the venom of various snakes. Hc-cath, a cathelicidin peptide derived from the venom of the sea snake *Hydrophis cyanocintus*, is of particular interest as it has been recently shown to display a broad range of antibiotic activity against clinical bacterial isolates^11^. Given our interest in developing new antibiotic treatments for CF patients, in this study we assessed the ability of Hc-cath to alleviate infection and inflammation in mouse models of infection with a view to evaluating this peptide as a candidate for therapeutic application.

## Results

### Sequence and physiochemical properties of Hc-cath

**Figure 1:**
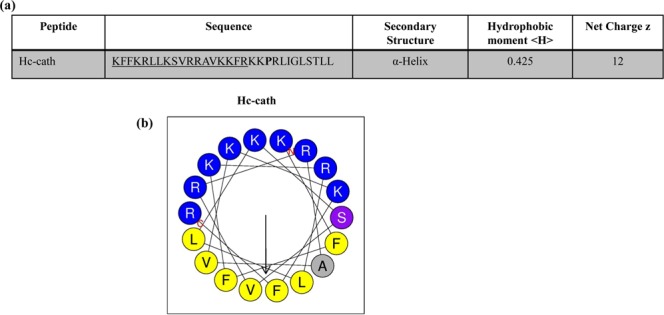
Analysis of Hc-cath primary and secondary structure. (**a**) Hc-cath hydrophobic moment and net charge. (**b**) Helical wheel projections demonstrate that the sequences shown in b possess distinct amphipathic structures with hydrophobic residues (yellow) collecting on one face of the helices and charged residues (blue) collecting on the opposite side.

The PSIPRED server and HeliQuest (www.expasy.org) predicted that Hc-cath has the capacity to form an alpha-helical structure, which is consistent with earlier reports^11^. HeliQuest was used to model the alpha-helical structure of Hc-cath demonstrating that Hc-cath displayed a hydrophobic moment of 0.425 and an overall charge of +12 (Fig. 1a). Hc-cath also forms an amphipathic helix with a high concentration of positively-charged residues on the polar face, the amphipathic helix is disrupted by the presence of a proline residue that is followed by a C-terminal disordered region (PRLIGLSTLL in Hc-Cath) (Fig. 1b).

### Hc-cath reduces LPS-induced inflammatory responses by macrophages

**Figure 2:**
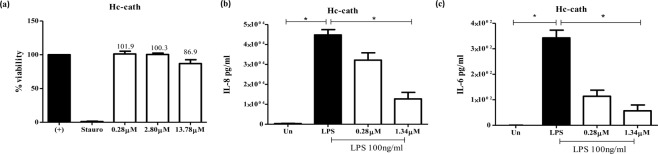
Hc-cath limits an LPS induced inflammatory response from macrophages. THP-1 macrophages were stimulated with increasing concentrations of Hc-cath (**a**) (0.28 µM, 2.80 µM or 13.78 µM) or staurosporine or left untreated (un). Viability was determined after 16 hr. (**b**,**c**) ELISA analysis of IL-8 and IL-6 release from differentiated THP1 macrophages 16 hr after stimulation with *Pseudomonas* LPS (100 ng/ml) in combination with 0.28 µM or 1.34 µM Hc-cath. Data are presented as mean ± SEM. Means between groups (untreated vs LPS and LPS versus LPS + Hc-cath) were compared by Mann Whitney t-test. ^*^Indicates P < 0.05.

Hc-cath displayed limited cytotoxicity (Fig. 2a) towards macrophages. We therefore assessed the ability of the peptide to downregulate LPS-induced cytokine responses at concentrations of 0.28 μM and 1.34 μM. At these concentrations, Hc-cath (Fig. 2b,c) significantly reduced the release of LPS-induced IL-6 and IL-8 confirming the ability of the peptide to limit LPS-induced inflammation.

### Hc-cath displays antimicrobial activity against lung relevant pathogens

**Figure 3:**
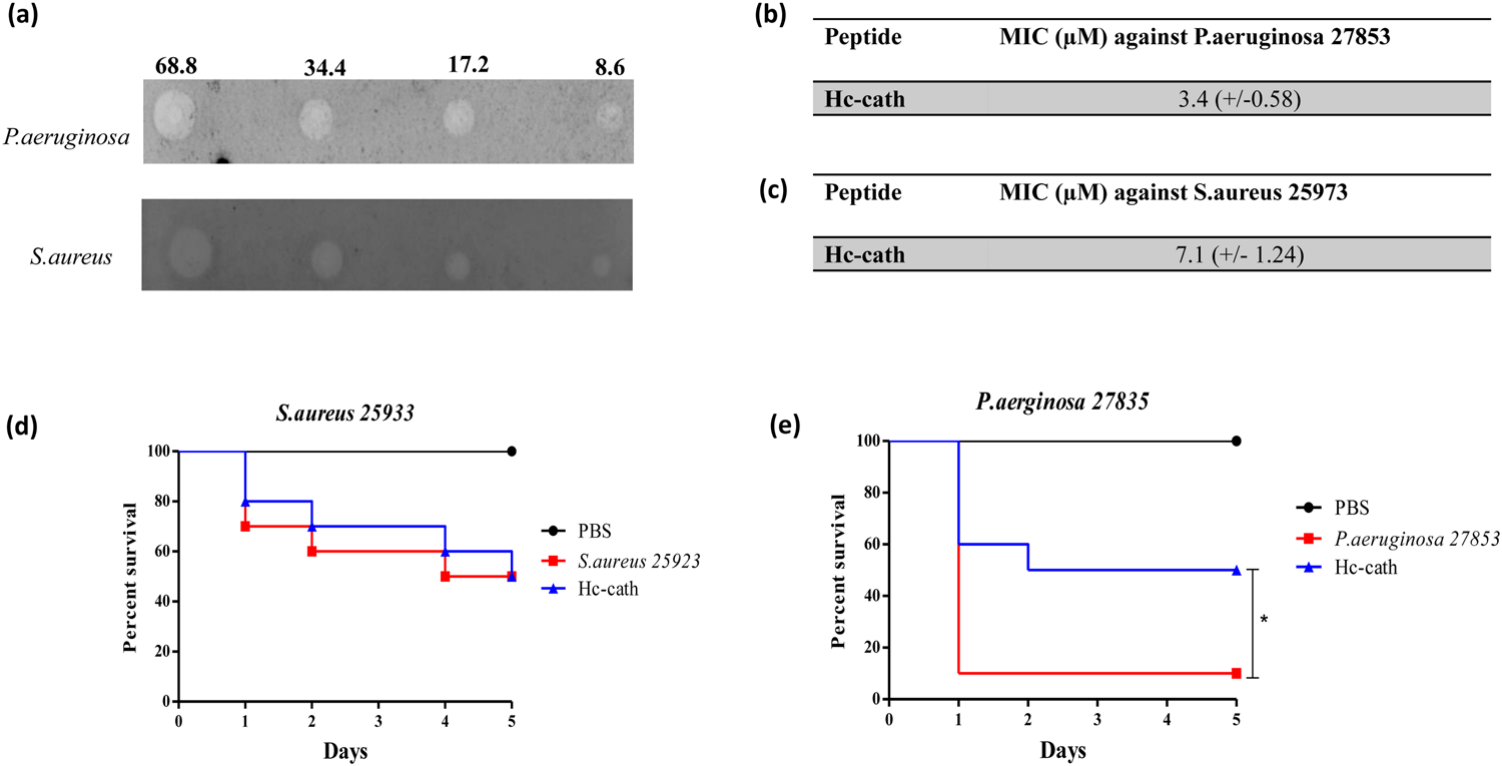
Hc-cath exerts an antimicrobial effect against *P*. *aeruginosa* and *S. aureus* and promotes Galleria survival. (**a**) Radial diffusion assay. Agarose gels were inoculated with 100 μl (OD_600_ − 0.4–0.5) of *P. aeruginosa* or *S. aureus* as indicated. Plates were challenged with 68.8 µM, 34.4 µM, 17.2 µM or 8.6 µM of Hc-cath and left overnight. Plates were stained with Coomassie blue overnight. Image presented representative of 3 independent experiments. (**b**,**c**) Minimum inhibitory concentration of Hc-cath vs (**b**) *P*. *aeruginosa* or (**c**) *S. aureus* as calculated from radial diffusion assays. Representative of 3 independent experiments. (**d**,**e**) Galleria survival over 5 days. Galleria were inoculated with 20 μl of *S. aureus* (OD_600_ – 0.25) or *P. aeruginosa* (OD_600_ – 0.1 – diluted 1 in 1,000,000) in PBS or PBS alone. Galleria received Hc-cath in 20 μl of PBS (68.8 μM) or a second injection of PBS. Galleria were incubated at 37 °C and survival was assessed every day. n = 10 galleria per group. Survival curves were compared using Kaplan-Meier log rank analysis. *Indicates P < 0.05.

We assessed the antimicrobial activity of Hc-cath against two lung-relevant pathogens, *P. aeruginosa* and *S. aureus*. Hc-cath demonstrated antimicrobial activity against both bacteria (Fig. 3a). Calculations of minimum inhibitory concentrations (MIC) revealed that Hc-cath was more potent against *P. aeruginosa* (Fig. 3b) compared to *S. aureus* (Fig. 3c)

We also investigated the efficacy of the peptide in an *in vivo* model of wax moth infection. *G. mellonella* were infected with *S. aureus* (Fig. 3d) or *P. aeruginosa* (Fig. 3e) before treatment with Hc-cath and survival assessed over a 5-day period. Hc-Cath did not influence the survival of *S. aureus* infected Galleria (Fig. 3d) compared to infection alone. *G. mellonella* were more sensitive to *P. aeruginosa* infection, as has been reported by others^12^, with 90% of Galleria succumbing to infection by day 5. However a significant improvement was observed in Galleria receiving Hc-cath (Fig. 3e).

### Hc-cath reduces the inflammatory burden in a mouse model of LPS induced acute lung inflammation

**Figure 4:**
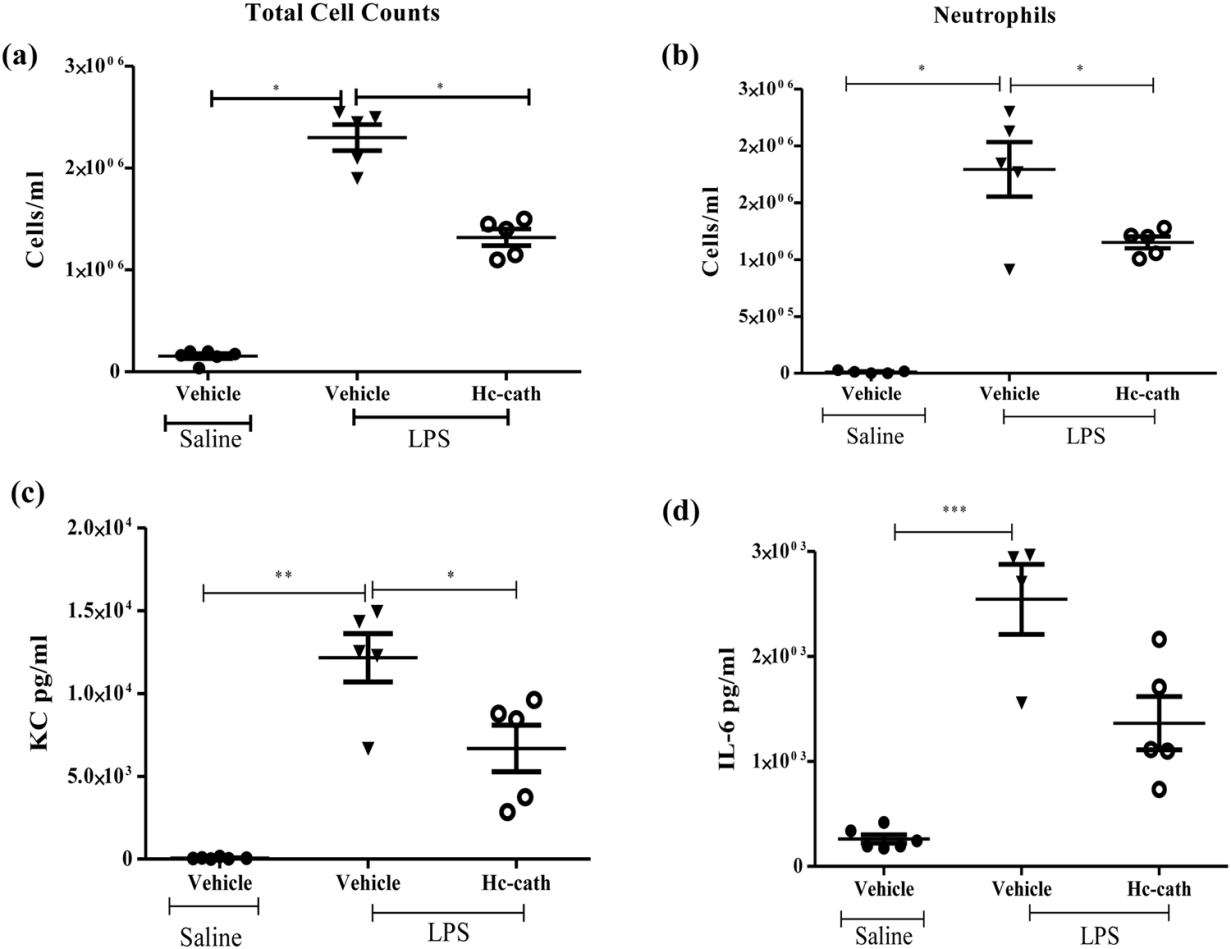
Hc-cath reduces the inflammatory burden in a mouse model of LPS induced acute lung inflammation. Age and sex matched female C57Bl6 mice received 100 μl of Hc-cath (137.8 μM) or vehicle (H_2_O) i.p. followed by a second dose after 24 hr. Mice then received 50 μl of LPS (0.4 mg/ml) via intra-tracheal instillation. Mice were sacrificed by overdose of rumpon-ketaset at 6 hr and bronchoalveolar lavage fluid was collected for analysis. Total cell counts (**a**) were obtained by enumeration by haemocytometer. Neutrophil (**b**) cell counts were obtained by staining of cytospins with May Grunwald and Geimsa stain and imaged using a Leica DM550 light microscope. ImageJ was using to quantify cell numbers. Release of KC (**c**) and IL-6 (**d**) into the BALF was measured by ELISA n = 3–6 mice per group. Data are presented as mean ± SEM. Means between groups (vehicle vs LPS and LPS versus LPS + Hc-cath) were compared by Mann Whitney t-test. *Indicates P < 0.05, **p < 0.01, ***p < 0.001.

Given the efficacy of Hc-cath in limiting LPS-induced inflammation in vitro (Fig. 2a–c), we evaluated the effects in an *in vivo* murine model of lung inflammation. As expected, a significant increase in bronchoalveolar lavage fluid (BALF) total cell counts was observed following LPS challenge (Fig. 4a), indicating the infiltration of immune cells into the lungs. This increase in total cells was significantly reduced in mice that received prior treatment with Hc-cath (Fig. 4a). Evaluation of differential cell counts demonstrated that neutrophils were the primary cell type infiltrating into the lungs (Fig. 4b). Hc-cath treatment significantly reduced neutrophil infiltration following LPS challenge as well as reducing associated inflammatory cytokines, KC (Fig. 4c) and IL-6 (Fig. 4d).

### Hc-cath treatment reduces the bacterial load and inflammatory burden in a mouse model of peritoneal infection

**Figure 5:**
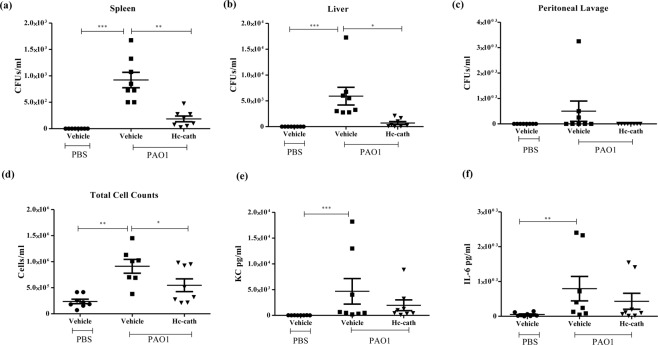
Hc-cath reduces the inflammatory burden and bacterial load in a mouse model of peritoneal infection. (**a**–**c**) Age and sex matched C57Bl6 mice received a 100 μl i.p. injection of Hc-cath (137.8 μM) or vehicle (H_2_O), followed by a second dose at 24 hr. Mice received 100ul PA01 (17.5 × 10^6^ CFU/mouse) via i.p. injection. Mice were culled after 6 hr by anaesthetic overdose. Peritoneal lavage fluid, spleen and liver were collected for analysis of bacterial load. Livers were homogenised in 2 ml of sterile PBS using a Bertin Precellys Evolution bead homogeniser. Spleens were homogenized in 1 ml sterile PBS by a handheld homogenizer. Spleen homogenate (**a**), liver homogenate (**b**) and peritoneal lavage fluid (**c**) were serially diluted in sterile PBS and 20ul was plated onto cetrimide agar and bacteria allowed to grow overnight before counting. (**d**–**f**) Peritoneal lavage fluid was collected for analysis of cellular infiltration and cytokine release into the peritoneum. Total cell counts (**d**) were obtained by enumeration by haemocytometer. Release of KC (**e**) and IL-6 (**f**) were measured by ELISA. n = 7–8 mice per group. Data are presented as mean ± SEM. Means between groups (vehicle vs PAO1 and PAO1 versus PAO1 + Hc-cath) were compared by Mann Whitney t-test. ^*^Indicates P < 0.05, **p < 0.01, ***p < 0.001.

We utilised a mouse model of P. aeruginosa peritoneal infection to examine the effect of Hc-cath on the bacterial load 6 hr after infection. Mice receiving Hc-cath treatment demonstrated a significant reduction in the bacterial load in the spleen (Fig. 5a) and liver (Fig. 5b) but not peritoneal lavage fluid (PLF) (Fig. 5c). Assessment of the PLF total cell counts (Fig. 5d) and the release of cytokines KC (Fig. 5e) and IL-6 (Fig. 5f) revealed a significant increase in the total cells within the peritoneal cavity upon infection. Hc-cath was effective at limiting this increase, significantly reducing the total cell counts compared to the infection alone mice (Fig. 5d). However, we did not observe a significant reduction in KC (Fig. 5e) or IL-6 levels (Fig. 5f) following treatment with Hc-cath, although there was an indication of a downward trend with Hc-Cath in both cases.

### The effect Hc-cath treatment on bacterial load, inflammatory cell infiltration and cytokine release in a mouse model of respiratory infection

**Figure 6:**
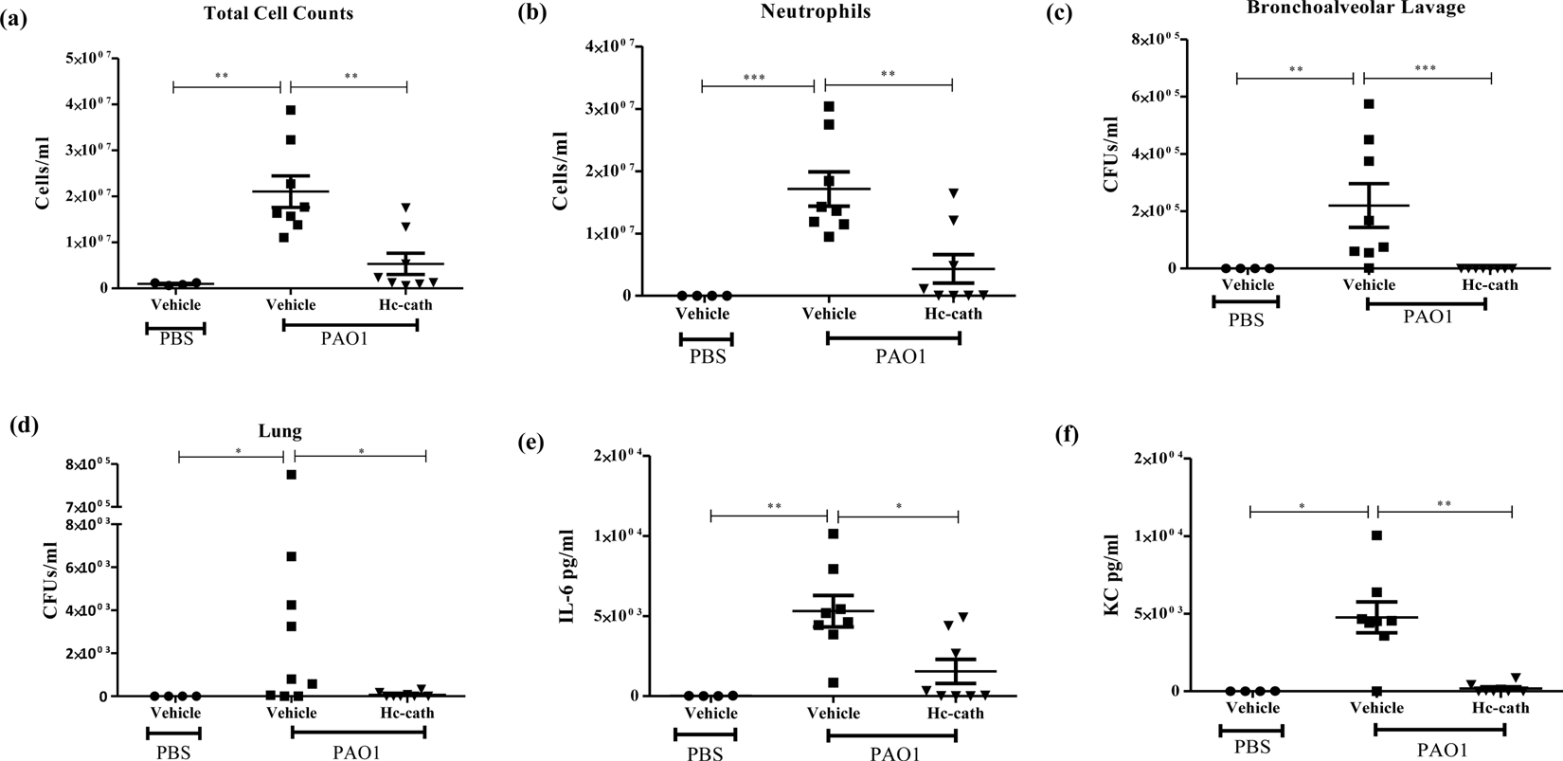
Intranasal delivery of Hc-cath reduces the cellular infiltration, bacterial load and cytokine release in a mouse model of respiratory infection. Age and sex matched C57Bl6 mice received Hc-cath (137.8 μM) in 30 μl plus 20 μl PBS or 50 μl PBS alone via intranasal delivery. After 24 hr mice received a second dose of 30 μl of Hc-cath (137.8 μM) plus 20 μl PAO1 in PBS (3.625–5.5 × 10^6^ per mouse) or PBS alone. Mice were culled at 6 hr with anaesthetic overdose and BALF was collected analysis of cellular infiltration. (**a**,**b**) Total cell counts (**a**) were obtain by enumeration by haemocytometer. Neutrophil (**b**) cell counts were obtained by staining of cytospins with May Grunwald and Geimsa stain and imaged using a Leica DM550 light microscope. Image J was using to quantify cell numbers. (**c**,**d**) BALF and lung homogenate were analysed for bacterial load. BALF was analysed for the release of IL-6 (**e**) and KC (**f**) by ELISA. ELISA. n = 7–8 mice per group. Data are presented as mean ± SEM. Means between groups (vehicle vs PAO1 and PAO1 versus PAO1 + Hc-cath) were compared by Mann Whitney t-test. *Indicates P < 0.05, **p < 0.01, ***p < 0.001.

As with the LPS instillation, introduction of *P. aeruginosa* into the lungs resulted in a significant increase in the total number of cells (Fig. 6a) in the lungs of which the majority were neutrophils (Fig. 6b). In mice receiving the peptide treatment we observed significant reduction in both these counts (Fig. 6a,b). Mice infected i.n. with *P. aeruginosa* PAO1 exhibited increased bacterial load in the BALF and the lungs after 6 hr (Fig. 6c). Pre-treatment with Hc-cath resulted in a significant reduction in bacterial load in BALF (Fig. 6c) and the lungs (Fig. 6d). Consistent with the reduction in infiltrating cells and bacterial burden there was a significant reduction in the release of inflammatory cytokines IL-6 (Fig. 6e) and KC (Fig. 6f) compared to infected mice.

## Discussion

With the increased incidence of antimicrobial resistance, there is a real need for new antibiotic agents. In this report, we have demonstrated the potent anti-inflammatory and antimicrobial activity of Hc-cath. As well as exhibiting limited cytotoxicity, Hc-cath demonstrated the dual ability of dampening inflammation and reducing infection.

The physiochemical properties of a peptide can give an indication of their activity and potency. The hydrophobic moment of peptides is an indicator of how strongly amphipathic the helix is^13^ and has been correlated with cytotoxic activity^14–17^ suggesting that the lower hydrophobic moment of Hc-cath which may contribute to the limited cytotoxic activity seen in Fig. 2a and has been shown Wei *et al.*^11^. Charge and the hydrophobic moment are also important factors for the antimicrobial activity of membrane active peptides such as these^17^, again with increasing charge and hydrophobic moment conferring greater membrane disruption. Despite this, Hc-cath demonstrated an good activity against *P. aeruginosa* and *S. aureus* as well improving survival in galleria (Fig. 3e) and reducing the bacterial burden in mice (Figs 5 and 6), which may suggest that its high charge is playing a more important role. Furthermore, from helical wheel analysis (Fig. 1a) it was found that a section (aa1-18) within the Hc-cath sequence formed a near empirical helical wheel, which has been shown to be important for bacterial membrane selectivity and disruption as has been observed for GKE21^18^ and the engineered α-helical peptide 7^19^, promoting the antimicrobial activity of the peptide. Together, one could speculate that the near empirical helices of Hc-cath promotes greater ability to select bacterial membranes and not to target host cells.

A protective effect of Hc-cath was seen in the models of Galleria *Pseudomonas* infection (Fig. 3e), highlighting its protective capacity *in-vivo*. However Hc-cath was not protective in Galleria models of *S. aureus* (Fig. 3d) infection and was generally less effective against *S. aureus*, which may possibly be due to cleavage by the *S. aureus* protease, aurolysin. Again the Galleria models highlight the lack of toxicity associated with Hc-cath as there was no impact of Galleria survival on Hc-cath administration.

Like LL-37, Hc-cath can bind LPS and prevent LPS signalling^11,20^, providing a probable mechanism for Hc-Cath’s anti-inflammatory activity in models of LPS induced inflammation *in-vitro*. This likely provides the mechanism of action of Hc-cath after LPS instillation (Fig. 4a,b) into the lungs, were Hc-cath significantly reduced cellular infiltration and cytokine release. However in the context of infection, Hc-cath displayed activity against lung relevant pathogens in the radial diffusion assays (Fig. 3a–c) of which activity against *P. aeruginosa* translated to *in-vivo* models (Figs 5 and 6).

Hc-cath reduced cellular infiltration in the peritoneum and bacterial load in spleen, liver and PLF when administered to mice i.p. However, despite a trend in reduction in IL-6 and KC, no significant effect was observed with Hc-cath treatment. It is possible that Hc-cath has a systemic antimicrobial effect thereby preventing spread to nearby organs. Hc-cath also reduced the recruitment of cells to the peritoneum, suggesting an ability to limit the inflammatory response in this compartment, which may be achieved via the improved clearance of bacteria as well as increased binding of LPS released by *P. aeruginosa*. Cath-2, a chicken cathelicidin, was shown to kill *Escherichia coli* in a manner that resulted in a significantly decreased inflammatory response when cultured together with macrophages compared to macrophages cultured with *E. coli* alone^21^. As well as improved anti-microbial activity stability and clearance are also worth considering as a factor contributing to the actvtiy of Hc-cath. Peptides tend to have a short half-life *in vivo* with enzymatic cleavage occurring in blood, liver and kidneys and peptides are excreted via the renal system^22^. The mechanism of clearance of Hc-cath is unknown, but this peptide is stable in serum for up to 6 hr^11^. Cleavage of LL-37 by neutrophil elastase and cysteinyl cathepsin proteases in the chronically inflamed lung has previously been shown^10^ and is an important consideration of any peptide therapeutic. The previously demonstrated stability of Hc-cath^11^ and the activity of Hc-cath with the more physiologically relevant environments within the mouse models highlight Hc-cath’s stability and activity.

Following i.n. administration, Hc-cath reduced the bacterial burden, neutrophil infiltration into the lungs and cytokine release. Again, this may relate to enhanced stability and greater activity of Hc-cath in the airways. *Bals et al*.^23^, demonstrated an antimicrobial effect following administration of an LL-37 over-expressing plasmid to the rodent lungs. This results appears to be similar to what we have demonstrated with Hc-cath. Others found that i.n. delivery of LL-37 with *P. aeruginosa* increased neutrophil infiltration after 6 hr, which was associated with a reduction in the bacterial load in the lungs^24^. However, in contrast, no increase in neutrophil infiltration was seen after Hc-Cath administration to the lungs during infection. This data would suggest Hc-cath is not chemotactic for neutrophils and that the antimicrobial activity of Hc-cath is via a direct action of the peptide on the bacteria. This is important to consider as neutrophils can be mediators of pathology in conditions such as CF, where reducing bacteria load without inducing further neutrophil infiltration may be of benefit.

Our studies suggest that Hc-cath has a potent antimicrobial effect against *P. aeruginosa* within the mouse lung. Importantly, as demonstrated in the peritoneal infection model, the antimicrobial activity of the peptide does not result in an inflammatory response due to the release of immunogenic components from dying bacteria. Cath 2 from chickens also does not cause inflammation from dying bacteria whereas such a side-effect was observed in mice treated with gentamicin^25^.

In conclusion, we have demonstrated the potential for Hc-cath to diminish infection and inflammation in pre-clinical mouse models. Given the need for new antimicrobials, Hc-cath is a potentially promising option to take forward into development as a therapeutic to combat infection.

## Materials and Methods

### Peptides

Hc-cath was synthesised by GL Biochem (Shanghai, China), respectively.

### Sequence and physiochemical properties of Hc-cath

Helical wheel projections were generated using HeliQuest server accessible via http://heliquest.ipmc.cnrs.fr. Primary amino acid sequences were submitted to the server and analysis parameters set to alpha helical with 18 amino acid analysis windows.

### THP-1 cell culture

Human acute monocytic leukemia cells (THP-1) were obtained from the American Type Culture Collection (ATCC, Manassas, USA) and were maintained in RPMI 1640 media (ThermoFisher Scientific, UK) supplemented with 10% heat-inactivated FCS (ThermoFisher Scientific, UK) and 1% Penicillin/Streptomycin (ThermoFisher Scientific, UK). For differentiation, phorbol 12-myristate 13-acetate (100 ng/ml; Sigma-Aldrich, Dorset, UK) was added to THP-1 cell suspension and cells were incubated for 72 hr period followed by 24 hr rest in fresh media. For LPS stimulation experiments, THP-1 macrophages were stimulated with *P. aeruginosa* LPS alone (100 ng/ml; Serotype 10, Source strain ATCC 27316; Sigma-Aldrich) or LPS in combination with peptide and incubated for 16 hr. Supernatants were collected for analysis by ELISA. For cell viability assays, THP-1 macrophages were incubated with Fluorofire-Blue ProViaTox kit (Molecutools, Dublin, Ireland) as per manufacturer’s instructions. Staurosporine (Sigma Aldrich, 10 mM) was used as positive control.

### Radial diffusion assays (RDA)

Radial diffusion assays (RDA) for *Pseudomonas aeruginosa* ATCC 27853 and *Staphylococcus aureus* ATCC 25923 were performed as previously described^26^.

### *Galleria mellonella* model of microbial infection

*S. aureus* and *P. aeruginosa* were grown aerobically to mid-logarithmic phase in Mueller Hinton Broth (Biokar Diagnostics, Beauvais, France) at 37 °C. The OD_600_ was adjusted to in-house optimised values of OD_600_ = 0.25 in sterile ice cold PBS (ThermoFisher Scientific, UK) for *S. aureus* and OD_600_ = 0.1, which was further diluted 1:1,000,000 in sterile PBS, for *P. aeruginosa*. Peptide in PBS (68.8 μM) or PBS alone was injected into the rear left proleg of each Galleria (Waxworms, UK), followed by 20 μl bacterial suspension via the rear right proleg. Uninfected controls received 2 × 20 μl injections of peptide or PBS. *G. mellonella* were incubated at 37 °C for 5 days and survival evaluated by onset of melanisation and movement in response to stimulus. Whole wax moth larvae were homogenised in 1 ml sterile PBS plated on blood agar after dilution for colony forming unit (CFU) viable counts.

### Mouse models of acute lung inflammation and infection

All experimentation was carried out in accordance with the Animal (Scientific Procedures) Act 1986 and current guidelines approved by the Queen’s University Ethical Review Committee. C57Bl6 mice (10–12 weeks of age) were used in all experiments and were purchased from Charles Rivers Laboratories (UK).

#### Mouse model of endotoxin-induced acute lung inflammation

Mice were administered Hc-Cath (137.8 μM) or vehicle (H_2_0) intraperitoneally (i.p.) every 24 hr for 2 days and challenged with Pseudomonas LPS (0.4 mg/ml; Sigma Aldrich) or saline alone intratacheally (i.t.) as previously described^27^. The mice were sacrificed 6 hr later, bronchoalveolar lavage fluid (BALF) collected for ELISAs and cell counts. Total cells counts were performed using a haemocytometer. Differential cell counts were enumerated (at least 400 cells per slide) on cytospins stained with May-Grünwald Giemsa (VWR, UK) imaged using a Leica DM55 brightfield microscope (Leica Microsystems, UK).

#### Intraperitoneal Pseudomonas aeruginosa infection

Mice were treated with Hc-cath (137.8 μM) or vehicle (H_2_O) as outlined above and challenged with *P. aeruginosa* (PA01, OD_600_ = 0.5 per mouse) or PBS alone delivered i.p. Mice were sacrificed 6 hr later and the peritoneal cavity was washed with 5 ml PBS and peritoneal lavage fluid (PLF) collected and used for the analysis for total cell counts and ELISA. Spleens were homogenised in 1 ml sterile PBS and livers were homogenised in 2 ml of sterile PBS for CFU counts. PLF, spleen homogenates and liver homogenates were diluted and plated on cetrimide agar for CFU counts.

#### Respiratory Pseudomonas aeruginosa infection

Mice received Hc-cath (137.8 μM) in 30 μl plus 20 μl PBS or 50 μl PBS alone via intranasal delivery. After 24 hr mice received a second dose of 30 μl of Hc-cath (137.8 μM) plus 20 μl PAO1 in PBS (OD. 0.5) or PBS alone. All i.n. instillations were carried out during anaesthetisation using isoflurane. Mice were sacrificed 6 hr later and BALF collected and processed as described above. Lungs and spleen were collected, homogenised in 1 ml of sterile PBS, and plated on cetrimide agar for CFU counts.

### ELISAs

Levels of IL-6 (Thermo Fisher Scientific, UK) and IL-8 (Biolegend, London, UK) in cell-free THP-1 macrophage supernatants and KC (R&D Systems, Abingdon, UK) and IL-6 (Thermo Fisher Scientific, UK) in cell-free BALF were quantified by ELISA as per manufacturer’s instructions.

### Statistics

All data were analysed using GraphPad Prism 5.0 (GraphPad Software Inc., San Diego, CA). Data are presented as mean ± SEM. Means were compared by Mann Whitney t-test where vehicle/untreated or drug (Hc-cath) group data was compared to LPS or infection (PAO1) data. P < 0.05 was accepted to indicate statistical significance. Survival curves were compared using Kaplan-Meier log rank analysis. *Indicates P < 0.05, **p < 0.01, ***p < 0.001.

## Data Availability

The datasets generated during and/or analysed during the current study are available from the corresponding author on reasonable request.
